# Reliability of Single-Use PEEP-Valves Attached to Self-Inflating Bags during Manual Ventilation of Neonates – An In Vitro Study

**DOI:** 10.1371/journal.pone.0150224

**Published:** 2016-02-25

**Authors:** Julia C. Hartung, Silke Wilitzki, Marta Thio-Lluch, Arjan B. te Pas, Gerd Schmalisch, Charles C. Roehr

**Affiliations:** 1 Department of Neonatology, Charité University Medical Center, Berlin, Germany; 2 Department of Neonatology, The Royal Women’s Hospital, Parkville, Victoria, Australia; 3 Department of Neonatology, Leiden University Medical Center, Leiden, The Netherlands; 4 Newborn Services, John Radcliffe Hospital, Oxford University, Oxford, United Kingdom; 5 The Ritchie Centre, Hudson Institute for Medical Research, Monash University, Melbourne, Australia; Icahn School of Medicine at Mount Sinai, ARGENTINA

## Abstract

**Introduction:**

International resuscitation guidelines suggest to use positive end-expiratory pressure (PEEP) during manual ventilation of neonates. Aim of our study was to test the reliability of self-inflating bags (SIB) with single-use PEEP valves regarding PEEP delivery and the effect of different peak inflation pressures (PIP) and ventilation rates (VR) on the delivered PEEP.

**Methods:**

Ten new single-use PEEP valves from 5 manufacturers were tested by ventilating an intubated 1kg neonatal manikin containing a lung model with a SIB that was actuated by an electromechanical plunger device. Standard settings: PIP 20cmH_2_O, VR 60/min, flow 8L/min. PEEP settings of 5 and 10cmH_2_O were studied. A second test was conducted with settings of PIP 40cmH_2_O and VR 40/min. The delivered PEEP was measured by a respiratory function monitor (CO_2_SMO^+^).

**Results:**

Valves from one manufacturer delivered no relevant PEEP and were excluded. The remaining valves showed a continuous decay of the delivered pressure during expiration. The median (25^th^ and 75^th^ percentile) delivered PEEP with standard settings was 3.4(2.7–3.8)cmH_2_O when set to 5cmH_2_O and 6.1(4.9–7.1)cmH_2_O when set to 10cmH_2_O. Increasing the PIP from 20 to 40 cmH_2_O led to a median (25^th^ and 75^th^ percentile) decrease in PEEP to 2.3(1.8–2.7)cmH_2_O and 4.3(3.2–4.8)cmH_2_O; changing VR from 60 to 40/min led to a PEEP decrease to 2.8(2.1–3.3)cmH_2_O and 5.0(3.5–6.2)cmH_2_O for both PEEP settings.

**Conclusion:**

Single-use PEEP valves do not reliably deliver the set PEEP. PIP and VR have an effect on the delivered PEEP. Operators should be aware of these limitations when manually ventilating neonates.

## Introduction

Self-inflating bags (SIB) are widely used for neonatal resuscitation. They are the most commonly used devices for manual ventilation of neonates in several countries.[1, 2] Perceived advantages of SIBs are: 1) their comparatively low cost; 2) their ease of use; 3) they can be used independently of a gas source, i.e. outside the hospital or in resource-limited settings;[3, 4] 4) they can be used with a gas source attached to deliver higher oxygen concentrations; 5) they can provide positive end-expiratory pressure (PEEP) when used in conjunction with additional PEEP valves. Several animal studies demonstrated the benefits of applying PEEP during ventilation: PEEP helps establish and maintain functional residual capacity,[5, 6] which is essential during transition to extrauterine life.[7, 8] Therefore, recent resuscitation guidelines suggest the use of PEEP for resuscitation of preterm newborn infants.[9, 10] However, studies by our group as well as others have found that PEEP valves often do not reliably deliver the PEEP as intended.[11–13] In a recent study we tested 10 factory new multi-use PEEP valves throughout 30 cycles of thermo-sterilization and demonstrated that these procedures further decreased their reliability.[14]

Consequently, SIBs with single-use PEEP valves might represent a more reliable alternative than SIBs with multi-use PEEP valves. However, single-use PEEP valves have not been well studied and very little is known about their ability to generate the set PEEP. Therefore, the aim of our study was to test the reliability of single-use PEEP valves from different manufacturers during simulated resuscitation of preterm infants in the delivery room and to investigate the effect of peak inflation pressure (PIP) and ventilation rate (VR) settings on the delivered PEEP.

## Material and Methods

We tested 10 factory new single-use PEEP valves from 5 different manufacturers: two valves each from Ambu^®^ (0–20 cmH_2_O; Ballerup, Denmark), DROH^®^ (0–10 cmH_2_O; Mainz, Germany), Medisize^®^ (5–20 cmH_2_O; Hillegom, Netherlands), The Bag II^®^ (5–20 cmH_2_O; Laerdal Medical, Stavanger, Norway) and Vital Signs Inc. ^®^ (5–20 cmH_2_O; Totowa, NJ, USA). All had a 30 mm connection and a freely adjustable spring valve for setting the PEEP to the above given range.

**Fig 1:**
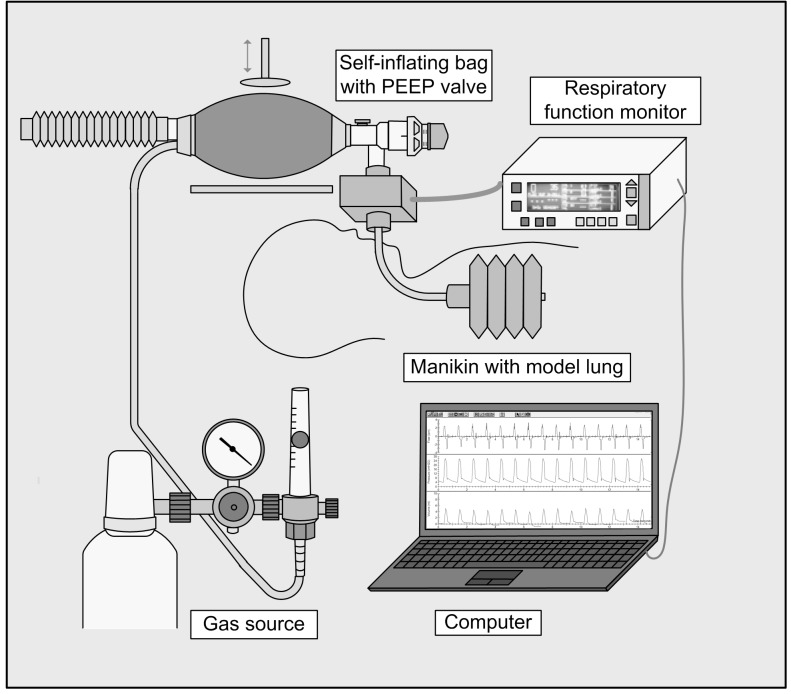
Schematic diagram of the experimental setup: The single-use PEEP valves were consecutively attached to a mechanically driven self-inflating bag. A gas source was connected. Subsequently a manikin simulating a 1kg neonate was ventilated. The delivered pressures and volume were measured using a respiratory function monitor and analyzed with a laptop computer. Standard settings were: Peak inspiratory pressure = 20 cmH_2_O, ventilation rate = 60/min, flow = 8 l/min.

The valves were attached to a new disposable Ambu SPUR II Baby Resuscitator (Ambu^®^, Ballerup, Denmark) with a maximum stroke volume of 150 ml that was designed to ventilate neonates and infants of up to 10 kg bodyweight. The bag had a pressure-relief valve set at 40 cmH_2_O. An oxygen reservoir tube with a volume of approximately 100 ml was attached and the bag was connected to medical gas (Flow 8 l/min, FiO_2_ 0.21). An intubated, leak free neonatal manikin (Fisher & Paykel Healthcare^®^, Auckland, New Zealand) simulating a 1kg neonate, that contained a neonatal lung model with a compliance of 2.0 ml*kPa^-1^ was ventilated. Airway pressure, gas flow and volume were detected using a CO_2_SMO^+^ PLUS! monitor (Novametrix Medical Systems^®^, Wallingford, CT, USA). The experimental setup is shown in Fig 1. Data were recorded by a laptop computer using the CO_2_SMO^+^ Software (for Windows, Novametrix, version 1.0).

**Fig 2:**
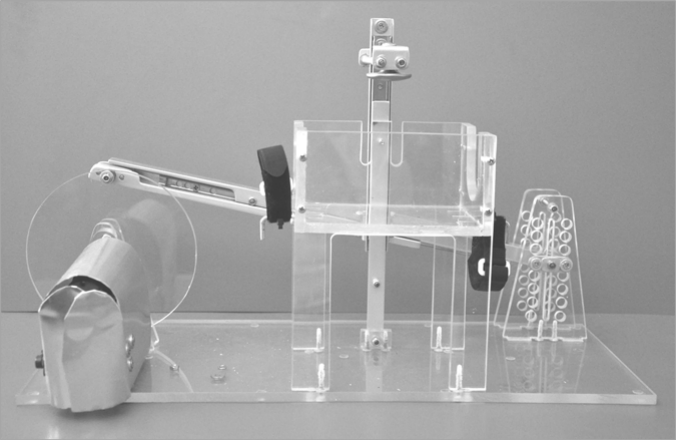
Electromechanical device constructed to compress the SIB under standardized conditions.

We constructed an electromechanical device that allowed compression of the SIB under standardized laboratory conditions (Fig 2). Positive inspiratory pressure (PIP) could be set by modifying the indentation depth of a plunger compressing the bag. The device was actuated by an electrical engine that was connected to an adjustable power supply. The ventilation rate (VR) could be set by altering the motor voltage.

Standard settings of PIP = 20 cmH_2_O, gas flow = 8 l/min, VR = 60 breaths per minute and PEEP = 5 and 10 cmH_2_O were used and extended by measurements with PIP = 40 cmH_2_O and VR = 40 /min to study the effect of PIP and VR on the delivered PEEP. PIP was adjusted mechanically without the PEEP valve attached (indentation depth of the plunger) and VR was adjusted by the motor voltage according to the measured and displayed PIP and VR values of the CO_2_SMO^+^. The device was set before the valves were attached to guarantee equal conditions for all the measurements. The adjustment of PIP = 20 cmH_2_O could be performed without any problems, however, after attachment of the PEEP valve there was a slight increase of the PIP: The median PIP (25^th^ and 75^th^ percentile) was 26.3. (25.0–27.6), 26.0 (24.5–27.5), 20.8 (20.0–22.2), 25.3 (23.8–26.9), 25.4 (23.7–27.1) cmH_2_O for valves of manufacturer 1–5. For a set PIP of 40 cmH_2_O the targeted value could not be reached during most measurements due to early opening of the pressure relief valve. The median PIP (25^th^ and 75^th^ percentile) for manufacturer 1–5 was 37.4 (37.2–37.6), 37.4 (37.3–37.5), 37.1 (37.0–37.2), 37.3 (37.3–37.4), 37.4 (37.3–37.5) cmH_2_O. VR during the measurements showed no relevant deviations from the set values. Eight 30-second-measurements with the different combinations of parameter settings were performed with each valve and repeated 5 times to test reproducibility of our data (adding up to 400 measurements in total). PEEP was defined as the pressure at the end of the expiratory phase, just before the start of the subsequent inflation and was calculated by the software of the CO_2_SMO^+^ respiratory function monitor. Ten consecutive inflation cycles of each measurement were evaluated and the median PEEP was calculated. Because there was no statistically significant difference in the delivered PEEP between both valves of the same manufacturer, the data were pooled.

All measured PEEP values are presented as medians with 25^th^ and 75^th^ percentile in brackets and were compared using the Mann-Whitney or Kruskal-Wallis test, as appropriate. For each parameter setting the reproducibility of serial PEEP values during the 30-second-measurements was described by coefficient of variation (CV [%] = 100*SD / mean) of 10 consecutive inflation cycles and compared using the Kruskal-Wallis test. The effect of changes in PIP and VR on the delivered PEEP was tested separately by the Wilcoxon test for paired samples. The relationship between the decrease of the delivered PEEP and the increase of the delivered volume was investigated by linear regression analysis. Statistical analysis was performed using Statgraphics Centurion® software (Version 16.0, Statpoint Inc., Herndon, Virginia, USA) and MedCalc (Version 9.2.0.2; MedCalc Software, Mariakerke, Belgium). A p-value less than 0.05 was considered statistically significant.

## Results

### Reproducibility

The coefficient of variation of 10 consecutive inflations of each PEEP valve was calculated. Table 1 shows median and 25^th^ and 75^th^ percentile of 80 series of measurements (two valves of each manufacturer measured with 8 different combinations of parameter settings, repeated 5 times) per valve. As valve 3 did not deliver any relevant PEEP, it was excluded from the analysis. There were no statistically significant differences in the CVs between valves 1, 2 and 5. Only valve 4 showed a weak but statistically significantly (p<0.001) higher variability in the delivered PEEP. However, median CV of all remaining valves (except valve 3) was <2% and negligible from the clinical point of view, indicating highly reproducible PEEP during the individual measurements.

**Table 1:** Coefficient of variation.

	Valve 1	Valve 2	Valve 3	Valve 4	Valve 5	p-value
**CV**_**PEEP**_ (%)	1.12 (0.66–2.04)	1.36 (0.74–2.24)	**-**	1.89 (1.11–3.07)	1.08 (0–2.32)	**<0.001** (0.158)^1)^

^1)^without valve 4

Coefficient of variation (CV) of serial PEEP values of the PEEP valves of 5 manufacturers (Presented are median and 25^th^ and 75^th^ percentile in brackets of 80 series of measurements (two prototypes, 8 parameter settings, 5 repetitions) per valve. Statistically significant p-value is printed in bold)

### Delivered PEEP

**Fig 3:**
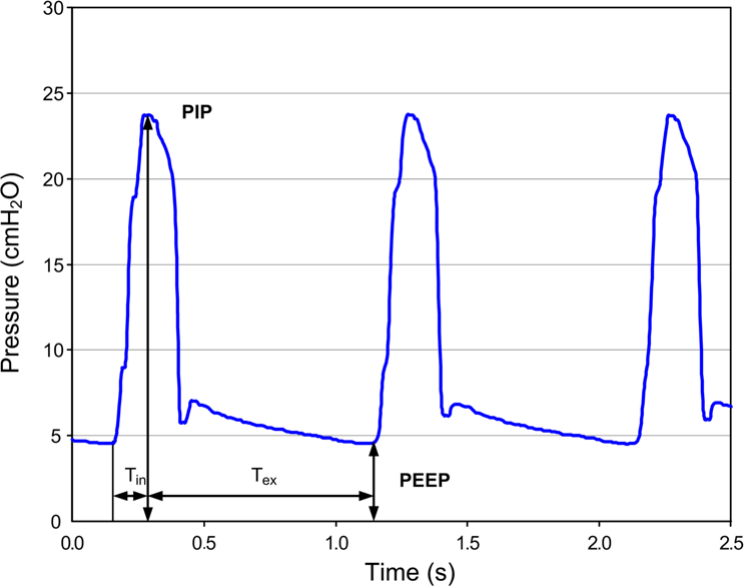
Pressure profile of a self-inflating bag with 8 l/min flow supply.

The pressure curves that were recorded for each inflation cycle showed a continuous decrease of the delivered pressure during the expiratory phase of the ventilation cycle until the final PEEP was reached just before the subsequent inflation started. This pressure profile is illustrated in Fig 3.

In contrast to the serial measurements, the variability of the delivered PEEP between the different parameter settings was significantly higher, as shown in Table 2. Again, valve 3 was excluded from the analysis because no relevant PEEP could be delivered. Only in three of the eight combinations of parameter settings statistically significant differences (p<0.05) in the delivered PEEP could be shown between the manufacturers. However, these differences were weak and near the limit of statistical significance.

**Table 2:** Delivered PEEP.

PEEP Setting	PIP Setting	VR Setting	PEEP measured (cmH_2_O)				p-value
(cmH_2_O)	(cmH_2_O)	(1/min)	Valve 1	Valve 2	Valve 4	Valve 5	
5	20	40	3.44 (2.81–4.17)	2.89 (2.15–3.12)	2.16 (1.85–2.89)	2.67 (2.34–3.28)	**0.049**
5	20	60	4.19 (3.41–4.80)	3.30 (3.14–3.73)	2.84 (2.36–3.66)	3.04 (2.56–3.50)	**0.021**
5	40	40	1.64 (1.09–1.91)	1.60 (1.52–1.95)	1.35 (1.13–1.71)	1.52 (1.47–1.65)	0.515
5	40	60	2.70 (1.19–3.00)	2.39 (2.30–2.63)	1.87 (1.62–2.24)	2.43 (2.30–2.59)	0.246
10	20	40	6.44 (4.39–7.91)	4.53 (3.26–6.11)	4.06 (3.31–5.21)	5.42 (3.46–6.33)	0.118
10	20	60	7.10 (5.20–8.63)	6.06 (5.10–6.33)	5.04 (3.60–5.66)	6.38 (4.81–7.34)	**0.039**
10	40	40	3.03 (1.88–3.52)	2.72 (2.40–4.00)	2.05 (1.38–3.10)	2.91 (2.55–4.48)	0.083
10	40	60	4.57 (2.34–5.29)	4.35 (4.29–4.63)	3.07 (2.89–3.28)	4.65 (4.11–4.90)	0.055

Comparison of the delivered PEEP by the resuscitation bag using 2 PEEP valves each of 5 different manufacturers and two parameter settings each for PEEP, peak inspiratory pressure (PIP) and ventilation rate (VR). Valve 3 did not generate any PEEP and was excluded from the evaluation. (Presented are median and 25^th^ and 75^th^ percentile in brackets. Statistically significant p-values are printed in bold).

The measured PEEP was lower than the preset PEEP for all parameter settings. For standard settings the median (25^th^ and 75^th^ percentile) PEEP was 3.4 (2.7–3.8) cmH_2_O and 6.1 (4.9 -7.1) cmH_2_O for a set PEEP of 5 and 10 cmH_2_O, respectively. Using the pooled data of all measurements the relative deviation from the set PEEP was only slightly higher for 10 cmH_2_O (-55.6% (-69.9%—-40.8%)) than for 5 cmH_2_O (-52.7% (-65.8%—-36.6%)). As shown in Table 2, the influence of PIP and VR on the delivered PEEP was distinctly higher than the differences between the manufacturers.

### Effect of PIP and VR

**Fig 4:**
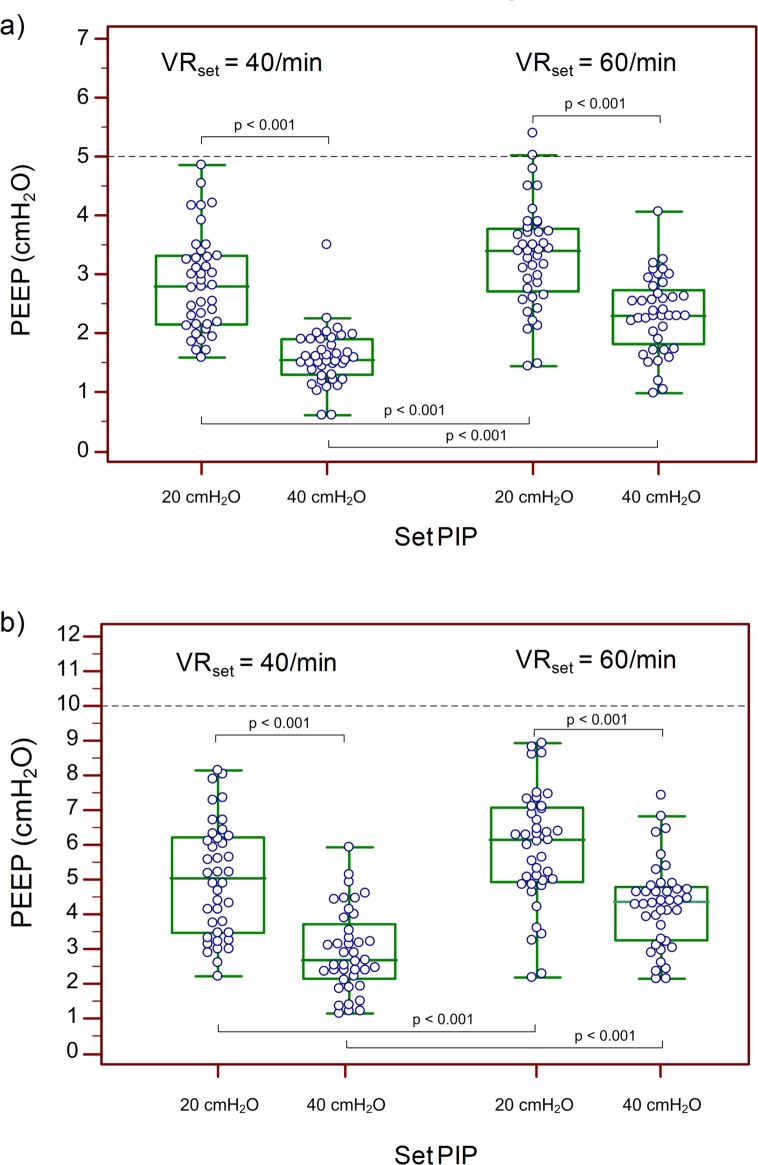
Effect of PIP and VR. Effect of PIP and VR on the delivered PEEP for a PEEP setting of a) 5 cmH_2_O and b) 10 cmH_2_O.

The effect of PIP and VR on the measured PEEP is shown in Fig 4, using pooled data of valves 1, 2, 4 and 5. For both PEEP settings, there was a statistically significant decrease (p<0.001) of the delivered PEEP with increasing PIP. An opposing trend was seen for VR. With increasing VR and a shortening of the expiratory time the delivered PEEP increased significantly (p<0.001). For standard settings an increase of the PIP from 20 to 40 cmH_2_O lead to a decrease (p<0.001) in PEEP to 2.3 (1.8–2.7) cmH_2_O and 4.3 (3.2–4.8) cmH_2_O; a decrease of VR from 60 to 40/min also lead to a PEEP decrease (p<0.05) to 2.8 (2.1–3.3) cmH_2_O and 5.0 (3.5–6.2) cmH_2_O for a set PEEP of 5 and 10 cmH_2_O, respectively.

### Delivered tidal volume

**Fig 5:**
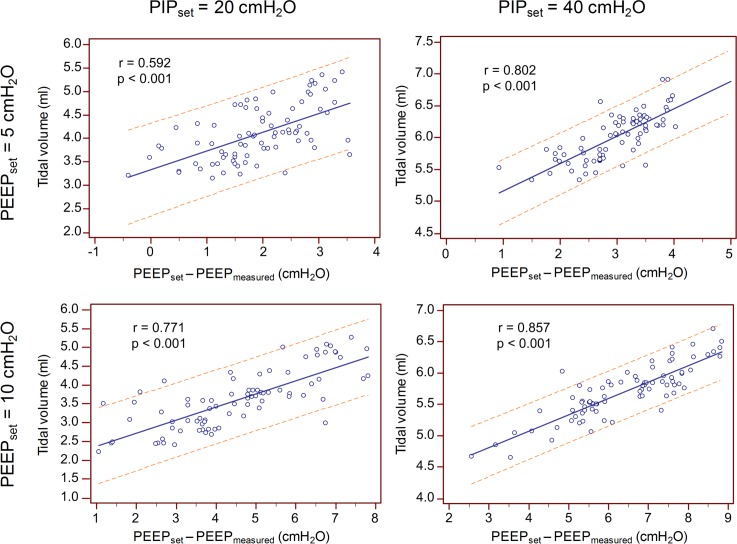
Effect of PEEP on tidal volume. Increase of the delivered tidal volume(V_t_) with increasing difference between the set and delivered PEEP for a PEEP setting of 5 cmH^2^O (top) and 10 cmH_2_O (bottom) and PIP of 20 cmH_2_O (left) and 40 cmH_2_O (right). Presented is the regression line with 95% prediction interval.

For both PIP and PEEP settings there was a statistically significant positive correlation between the delivered tidal volume (V_t_) and the difference between the set and the delivered PEEP (p<0.001; Fig 5). Hence, insufficient PEEP generation led to an increase of the delivered V_t_, as shown in Fig 5. The strength of this correlation increased with higher PIP and PEEP settings.

## Discussion

This in vitro study shows that the tested single-use PEEP valves do not reliably deliver the set PEEP and some valves did not generate any clinically relevant pressure. In contrast to T-piece resuscitators, the PEEP delivered by SIBs and PEEP valves decreased during the expiratory phase and the measured end-expiratory pressure was always lower than the set PEEP. The detected PEEP increased with increasing VR and decreased with increasing PIP. The impact of VR and PIP on the delivered PEEP was higher than the differences between manufactures.

### PEEP provision using a SIB

Several studies have shown that PEEP valves did not reliably deliver the set PEEP.[11–13] We have demonstrated in a previous study that even factory-new multi-use PEEP valves did not generate the PEEP to which they were set and repeated thermo-sterilization led to further functional impairment.[14]

In contrast to PEEP generation using T-piece resuscitators,[12, 15] during our measurements with the SIB and PEEP valve the pressure constantly decreased during the expiratory phase. This difference is due to the T-piece resuscitator being a constant flow device that can generate a continuous preset pressure. The driving flow can even compensate for small air leaks. Furthermore, the flow through the PEEP valve is nearly constant which improves the stability of PEEP generation. As opposed to that, using a SIB the remaining PEEP during expiration is influenced by the highly variable patient flow and even small air leaks (e.g. by the PEEP valve itself) can lead to a pressure decrease as visible in our study. However, T-piece devices cannot be operated without a constant gas flow, so in out-of-hospital settings or situations in which a gas flow is not readily available a SIB can be applied instead.

### Effect of PIP and VR on the delivered PEEP

PEEP provision was influenced by the VR, as previously shown by Morley et al.[13] They demonstrated that no sufficient PEEP could be delivered at low VRs, particularly at less than 40 inflations per minute.[13] In our study measurements with VR of 40 and 60 /min were conducted to demonstrate that higher VRs lead to more reliable PEEP provision. As mentioned above, PEEP decreases continuously during expiration with the SIB. Consequently, with lower VRs time between inflations extends and PEEP decreases to lower levels.

Unexpectedly, increasing PIP led to lower PEEP levels. We suppose that this is due to increasing leak flow at higher pressures. As the model lung and intratracheal tube were firmly connected and leak-free, we speculate, that there may have been an air leak in the SIB or PEEP valve. In a previous study we could demonstrate a significant decrease of PEEP in the presence of leak when using a SIB, despite the use of a PEEP valve.[16] Furthermore, the targeted PIP of 20 cmH_2_O was slightly exceeded as soon as a PEEP valve was attached and 40 cmH_2_O could not be reached during most of the measurements, as the pressure relief valve opened early and prevented a further rise of the pressure. This may have had an impact on PEEP as well.

### Effect of lower PEEP on delivered tidal volume

The delivered V_t_ during manual ventilation with a SIB depends on the compliance of the lung and the pressure difference between PIP and PEEP. In our experimental setup the compliance of the model lung was constant and PIP could be preset by adjustment of the indentation depth of the compressing device. Consequently, insufficient generation of PEEP increased the pressure difference and resulted in higher delivered V_t_. The impact of insufficient PEEP on V_t_ is illustrated in Fig 5. A similar effect could be observed in a previous study using multi-use PEEP valves, where increasing leak resulted in lower PEEP levels and consequently higher V_t_.[16] To which extent the demonstrated effects might be observed in vivo remains unclear, as we were unable to sufficiently simulate the changing respiratory mechanics of a preterm neonate in our laboratory setting.

### Clinical implications

The tested SIBs and PEEP valves could not deliver the set PEEP. Newborn infants suffering from respiratory distress may benefit from the provision of an adequate PEEP, as it can help them establish a functional residual capacity [5, 6, 17] and improves oxygenation.[17–19] Insufficient PEEP levels during resuscitation in the delivery room should cautiously be avoided as they unnecessarily impede establishment of a functional residual capacity and can lead to atelectrauma.[20–22] There is even evidence suggesting that insufficient PEEP provision can contribute to a higher incidence of bronchopulmonary dysplasia.[23] Despite the fact that single-use PEEP valves are not subject to damage caused by repeated thermo-sterilization procedures or material fatigue due to long-term use, they do not reliably deliver the set PEEP. Operators should be aware of their unreliable PEEP provision and test the valves before clinical use as described elsewhere.[14] They should also be aware that a lower PEEP will generate a higher V_T_, which can contribute to potentially dangerous lung overdistension[8, 20, 24] and even brain injury.[25] In order to deliver an adequate PEEP level, operators should bear the effect of different VR and PIP on the delivered PEEP in mind and adjust the PEEP valve as appropriate.

### Limitations

The experimental setup allowed for standardized PIP and VR settings during the measurements, so the effect of each particular PIP, VR, PEEP adjustment and PEEP valve could be studied separately. However, our study has several limitations. As the measurements were conducted in the described laboratory setting, several factors that may influence PEEP provision during a real resuscitation scenario could not be taken into consideration. Using an intubated and leak-free manikin, flow leak with an undesirable effect on PIP and PEEP could be prevented. However, the effect of potential flow leak that is common during initial resuscitation applying bag and mask ventilation could not be assessed. Furthermore, we used the same lung model for all the measurements, hence the effect of different respiratory mechanics due to various diseases, gestational ages or compliance changes during the first minutes after birth could not be studied. As only two valves per manufacturer were tested, we cannot make general assumptions regarding the valves’ quality or extrapolate our results to the valves of other manufacturers.

## Conclusion

In conclusion, the single-use PEEP valves tested in our study delivered less than the set PEEP with all combinations of set PIP, PEEP and VR. PEEP valves should be tested before clinical use and substituted if necessary. Awareness of the valves’ characteristics and influencing factors is crucial for the provision of adequate PEEP during manual ventilation of the neonate.

## Abbreviations

CV: coefficient of variation
PEEP: positive end expiratory pressure
PIP: positive inflation pressure
SD: standard deviation
SIB: self-inflating bag
VR: ventilation rate
V_t_: tidal volume
